# The Efficacy and Safety of Topiramate in the Prevention of Pediatric Migraine: An Update Meta-Analysis

**DOI:** 10.3389/fped.2020.00028

**Published:** 2020-02-27

**Authors:** Xinwei Wu, Yan Zhang, Mei Lu, Xiaolin Yu, Xiang Ye, Xingbang Wang, Peiyan Shan

**Affiliations:** ^1^Department of Geriatrics, Qilu Hospital of Shandong University, Jinan, China; ^2^Department of Gastroenterology, Qilu Hospital of Shandong University, Jinan, China

**Keywords:** topiramate, pediatric, migraine, prevention, meta-analysis

## Abstract

**Background:** Migraine is the most common acute primary headache in children and adolescents. In 2014, topiramate became the first preventive drug for migraine, approved by the Food and Drug Administration (FDA) for adolescents. This meta-analysis was aimed to evaluate the efficacy and safety of topiramate in the prevention of pediatric migraine.

**Methods:** We searched the PubMed, EMBASE, Cochrane Library, and Chinese National Knowledge Infrastructure (CNKI) databases up to June 2019 for eligible randomized controlled trials (RCTs). The primary outcomes were mean migraine days per month, ≥50% reduction rate, and Pediatric Migraine Disability Assessment Scale (PedMIDAS) scores. RevMan5.3 software was performed for statistical analysis.

**Results:** Overall, 5 RCTs recruiting 531 patients (6–17 years of age) were included in the meta-analysis. The target dose of topiramate was 2 mg/kg (the maintenance phase was 12 weeks), 2–3 mg/kg, 50 mg/day, and 100 mg/day (maintaining for 16 weeks), respectively, in the included studies. Our results demonstrate that participants receiving topiramate had a significant advantage in remitting the monthly migraine days than those receiving placebo, with a mean difference (MD) of −0.78 (*n* = 531; 95% CI, −1.23 to −0.32; *Z* = 3.37; *P* = 0.0008). Topiramate could also reduce the mean PedMIDAS scores (*n* = 238; 95% CI, −16.53 to −0.49; *Z* = 2.43; *P* = 0.04). However, there was no significant difference in the percentage of patients experiencing a ≥50% reduction in monthly headache days between topiramate and placebo groups (*n* = 531; 95% CI, 0.94–1.77; *Z* = 1.58; *P* = 0.11). Topiramate was associated with higher rates of side effects such as weight decrease (*n* = 395; 95% CI, 2.73–22.98; *Z* = 3.81; *P* < 0.01) and paresthesia (*n* = 531; 95% CI, 3.05–13.18; *Z* = 4.94; *P* < 0.01).

**Conclusions:** Topiramate can significantly decrease monthly headache days and migraine-related burden in migraine patients <18 years old. However, it failed to increase 50% response rate. Adverse events seem to be more frequent in topiramate-treated children.

## Introduction

Headache is the third cause of school absence among the pediatric population (1), and migraine is the most common acute primary headache in children and adolescents (2). Epidemiological studies have reported that migraine affects 3–5% of children, and the prevalence increases to 10–20% among adolescents (3–5). There is a slight male predominance before puberty; however, it is reversed after puberty (5). Unlike adults, pediatric migraine tends to manifest atypical clinical symptoms like episodic nausea, vomiting, nystagmus, vertigo, and so on (6).

Although ~20% of children with migraine can effectively get relieved before 25 years old, most of them still experience headache attacks through older ages (7). Pediatric migraine, which can affect the children's school performances and quality of life (8, 9), has become a significant problem for children. Most researchers (10) believe that if migraine has more than three to four episodes per month or the attack causes significant disability, which can be measured by the Pediatric Migraine Disability Assessment Scale (PedMIDAS) (11, 12), then preventive treatment for migraine needs to be initiated (13). Management of pediatric migraine includes treatment of acute headache attack and preventive treatment. The preventive treatment can be divided into pharmaceutical and non-pharmaceutical interventions (14). Drug treatment for pediatric migraine mainly consists of abortive and prophylactic medications.

Topiramate, an antiepileptic drug, which is widely used in the prevention of migraine in adults, was the first preventive drug approved by the Food and Drug Administration (FDA) for migraine in 12–18 years old adolescents (15). It is a neuromodulator with neuron-stabilizing properties (16), and its exact mechanism of effectiveness in migraine is unclear yet. Several randomized, double-blind trials have reported discordant results in the efficacy of topiramate for the pediatric migraine prevention, and these RCT trials have yielded disproportionate results (17, 18). In 2017, a meta-analysis (19) showed topiramate failed to decrease the monthly headache days or decrease the percentage of patients experiencing a ≥50% reduction in migraine days per month. However, the results seemed to be disputable because it had the following problems: (1) In one included study, topiramate was divided into two groups of 50 and 100 mg/day, so it was more reasonable to consider it as two RCT trials; and (2) the data of the meta-analysis were not accurate. For example, in the study of Powers et al., there were 66 patients in the placebo group, which was misclassified as 33 in the previous meta-analysis. To investigate whether topiramate treatment is beneficial compared to placebo for migraine prevention in children, we designed this meta-analysis of randomized controlled trials including four studies with a total of 531 patients.

## Methods

### Data Sources and Search Strategy

We searched the PubMed, EMBASE, Cochrane Library, and Chinese National Knowledge Infrastructure (CNKI) databases for eligible studies published up to June 2019 without language restrictions. The following keywords were used in our search strategies: (“topiramate” or “topamax”), AND (“pediatric migraine” or “pediatric headache” or “child/children/childhood migraine” or “child/children headache” or “adolescent/adolescents migraine” or “adolescent/ adolescents headache”). Conference abstracts, references of related studies, and reviews were also searched to avoid omitting relevant RCTs.

### Selection Criteria

The study was performed according to the Preferred Reporting Items for Systematic Reviews and Meta-Analyses (PRISMA) guidelines (20). Studies were considered eligible if they met the following criteria: (i) double-blind, randomized, and placebo-controlled trials that evaluated topiramate in migraine prevention; (ii) participants were children and adolescents (≤ 18 years old) with the clinical diagnosis of migraine according to the International Classification of Headache Disorders II (ICHD-II); and (iii) trials reported complete efficacy outcome. The exclusion criteria included reviews, animal trials, duplicate secondary analyses, studies comparing two or more interventions with each other but no contrast with placebo, and studies with incomplete or unavailable outcome data.

### Outcome Measures

According to the International Headache Society (IHS) recommendations (21), migraine days or days of migraine episodes were recommended as the primary efficacy outcomes. Headache index, intensity of headache, headache duration, and responder rates were used as the secondary evaluation for efficacy. In this study, mean migraine days per month post-treatment, ≥50% reduction rate, and PedMIDAS scores were extracted from the included literatures to estimate efficacy of topiramate treatment. When headache days was reported in some other unit of time, we adjusted all to be days of headaches per month. For feasibility analysis, it was assessed both by the proportion of patients who discontinued the study for any reason and by the proportion of patients dropout because of adverse effects.

### Data Extraction and Quality Assessment

Two experienced authors (Wu X. and Wang X.) screened the titles and abstracts of each literature independently to verify all potentially suitable trials that met the above inclusion criteria. Then, the study designs, participant characteristics, and outcomes were abstracted from the RCTs. Disagreements were resolved by discussion or following arbitration by the corresponding author. We used the “Risk of Bias” tool developed by the Cochrane Collaboration to assess the methodological quality of the trials.

### Data Analysis

We performed all statistical tests using RevMan5.3 software (Cochrane Information Management System). Continuous variables were analyzed with mean differences (MDs) along with 95% confidence intervals (CIs), and dichotomous outcomes were calculated of risk ratios (RRs) along with 95% CIs. Statistical significance was set at 0.10 for heterogeneity tests and 0.05 for all others. Heterogeneity was evaluated with *I*^2^. If *I*^2^ was >50%, heterogeneity of the enrolled trials was considered unacceptable and analyzed using random-effect model. If *I*^2^ was ≤ 50%, a fixed-effect model was chosen. RevMan5.3 software was performed for all statistical analysis. When there were more than 10 trials reporting the same outcome, the funnel plot analysis was used to evaluate publication bias.

## Results

### Search Findings

**Figure 1:**
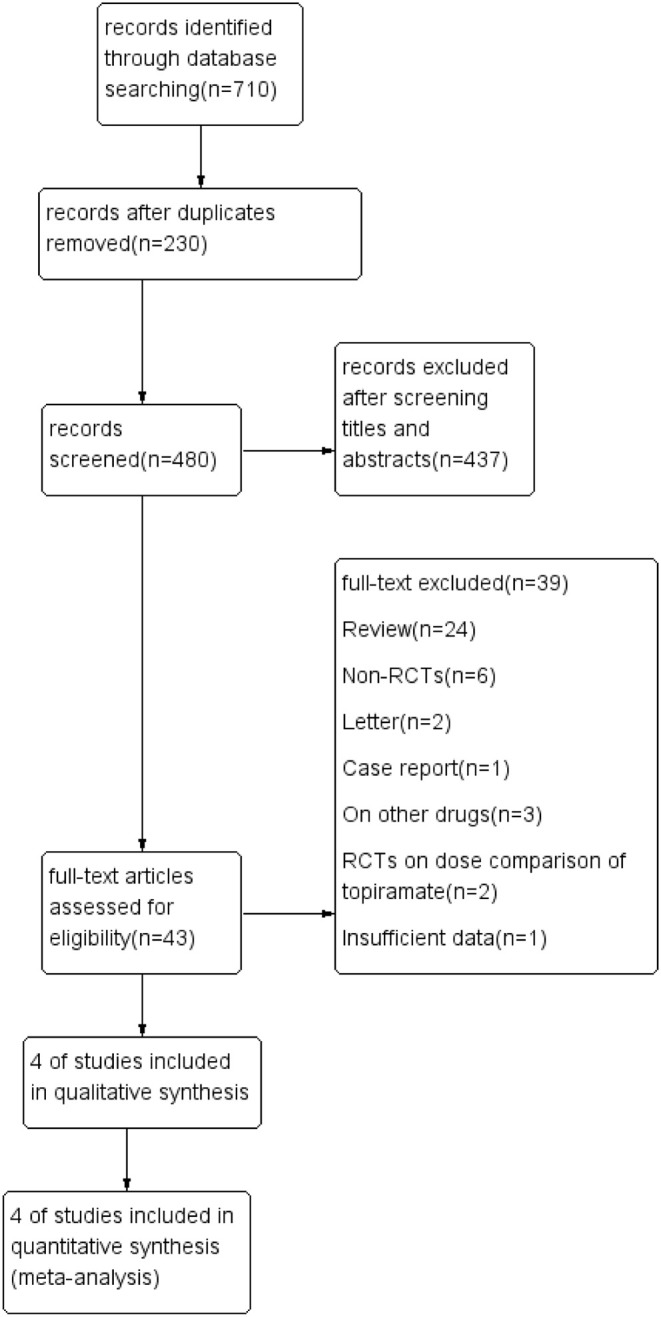
Study flow diagram.

Overall, 710 relevant articles were initially identified for the analysis, with 230 being duplicates resulting in exclusion. After screening the titles and abstracts of the remaining records, 437 papers were excluded. We reviewed 43 possibly relevant articles in full text, of which there were 24 reviews, 6 non-RCTs, 2 letters, and 1 case report, which were all excluded. In addition, two studies compared the efficacy between topiramate and propranolol, one study on topiramate and cinnarizine, along with two RCTs on dose comparison of topiramate, and one RCT did provide the precise outcome above even though it compared topiramate with placebo (Figure 1). At last, we identified four studies including five RCTs that met our inclusion criteria (Table 1).

**Table 1:** Characteristics of included randomized controlled trials.

**Study**	**Sample size**	**Mean age (years)**	**Girls (%)**	**Dropout (%)**	**Treatment groups**	**Dose range (mg/day)**	**Topiramate titration phase (week)**	**Topiramate maintenance phase (week)**	**Study design**	**Trial outcomes**
Powers et al. (22)	217	14.2 ± 2.4	69.1	9.7	Topiramate vs. placebo	2 mg/kg	8	16	Parallel	Mean headache days, reduction in monthly headache attacks, ≥50% responder rate, headache disability (PedMIDAS score)
Lakshmi et al. (23)	46	10.6 ± 1.4	31	8.7	Topiramate vs. placebo	100	4	12	Parallel	Mean headache days, ≥50% responder rate, reduction in mean migraine frequency and severity, headache disability (PedMIDAS score + school absenteeism), times of analgesics use
Winner et al. (24)	162	11.1 ± 2.5	48.4	19.1	Topiramate vs. placebo	2–3 mg/kg	8	12	Parallel	Mean headache days, reduction in monthly headache days, ≥50, ≥75, and 100% responder rate
Lewis et al. (25)	106	14.2 ± 1.6	61	17	Topiramate vs. placebo	50, 100	4	12	Parallel	Percent reduction in monthly migraine attack rate, mean headache days, ≥50% responder rate, rate of analgesics use

### Characteristics of Included Studies

Four papers containing 5 trials (22–25) recruiting 531 patients were included in the meta-analysis. The sample size in each study ranged from 46 to 217 (topiramate and placebo participants only), with one study (23) recruiting <50 patients. All the four papers reported the criteria for pediatric migraine diagnosis. The mean age of the study population was 12.5 years old, and 57.9% of the participants were girls. The predominant ethnic groups represented were Caucasian, as well as African, Asian, and others. One study (22) had three arms: topiramate, placebo, and a third treatment group—amitriptyline. The data of amitriptyline group was not included in this review. One of the studies included two dose treatments of topiramate (50 and 100 mg/day), and therefore, it was considered as two separate trials. All the included studies reported the duration of topiramate treatment ranging from 16 to 31 weeks. Washout and screening phases, weaning period, and follow-up were also incorporated into the studies. The dose of topiramate was gradually increased in all the included studies.

All the selected literatures reported days of headache and ≥50% reduction rate as trial outcomes. Two studies also reported difference in PedMIDAS scores between topiramate and placebo groups.

### Quality Assessment and Publication Bias

**Figure 2:**
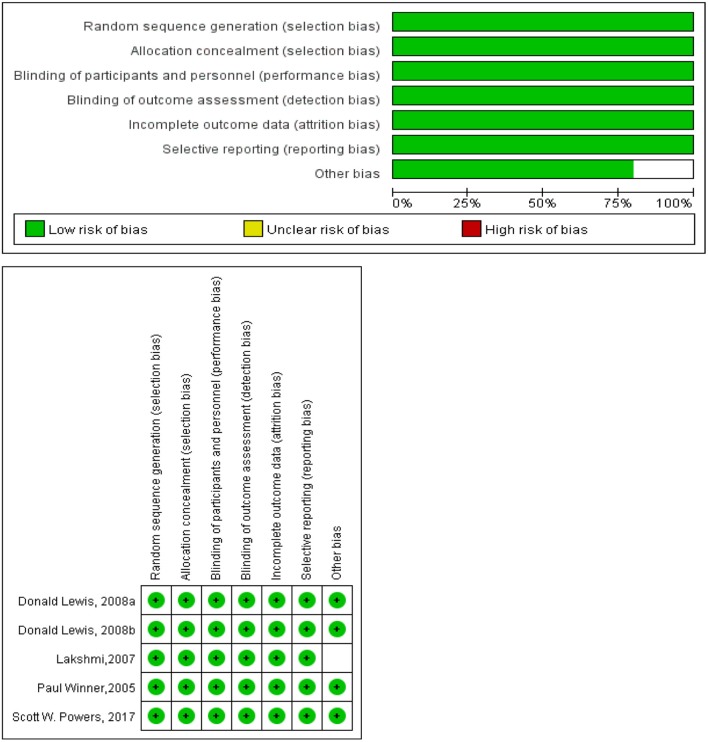
Risk of bias graph and summary.

The methodological quality of the trials was assessed by Cochrane Collaboration “Risk of Bias” tool. All the included trials described methods of random sequence generation and allocation concealment. Detailed information about blinding of participants and outcome assessment was reported in all studies. The outcome data were complete. The studies were at low risk of bias (Figure 2).

### Efficacy Outcomes

#### Primary Outcome

**Figure 3:**
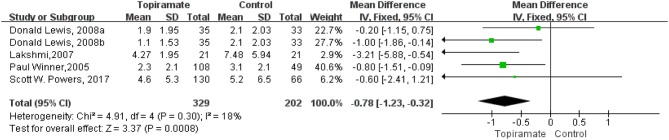
Comparison of the monthly migraine days between topiramate and placebo groups.

All the five selected trials reported monthly days of headache as a trial outcome. Our results demonstrated that participants receiving topiramate had a significant advantage in remitting the monthly migraine days than those receiving placebo, with an MD of −0.78 (*n* = 531; 95% CI, −1.23 to −0.32; *Z* = 3.37; *P* = 0.0008). The data selected in the analysis showed low heterogeneity (*I*^2^ = 18%; *P* = 0.30), and fixed-effects model was used. *Z* test for overall effect was statistically significant (*P* = 0.0008) (Figure 3).

#### Secondary Outcomes

**Figure 4:**
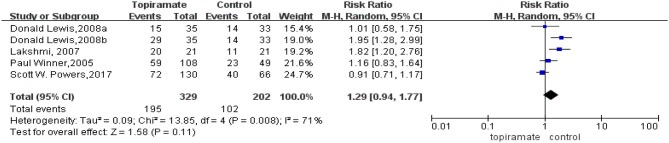
Comparison of ≥50% response rate monthly migraine days between topiramate and placebo groups.

**Figure 5:**
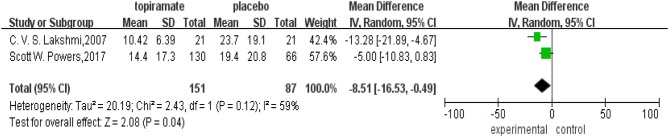
Comparison of Pediatric Migraine Disability Assessment Scale (PedMIDAS) scores between topiramate and placebo groups.

All the five trials included reported the rate of patients experiencing a ≥50% reduction in the number of headache days. This meta-analysis revealed that there was no significant difference in the percentage of patients experiencing a ≥50% reduction in monthly headache days between topiramate and placebo groups (*n* = 531; 95% CI, 0.94–1.77; *Z* = 1.58; *P* = 0.11). Random-effects model was used because the data showed heterogeneity (*I*^2^ = 71%; *P* = 0.003) (Figure 4). In addition, two studies reported headache-related disability as the outcome. Our results showed that there was significant difference between the two groups in the mean PedMIDAS scores (*n* = 238; 95% CI, −16.53 to −0.49; *Z* = 2.43; *P* = 0.04). The data selected in the analysis showed heterogeneity (*I*^2^ = 59%; *P* = 0.12), and random-effects model was used (Figure 5).

#### Side Effects and Adverse Reactions

**Figure 6:**
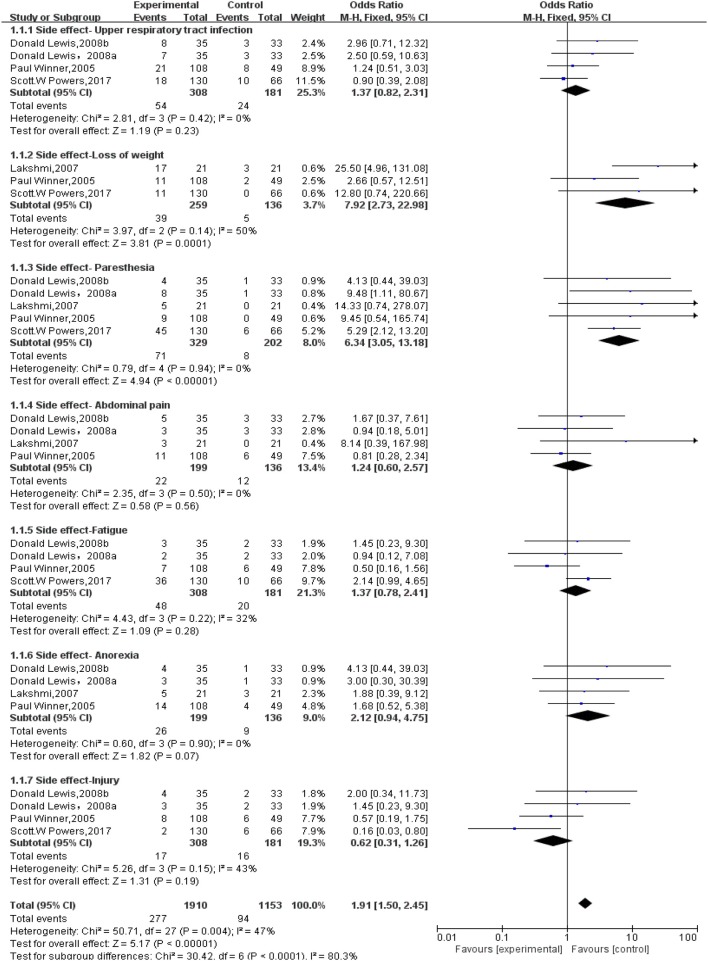
Comparison of side effects and adverse reactions between topiramate and placebo groups.

**Figure 7:**
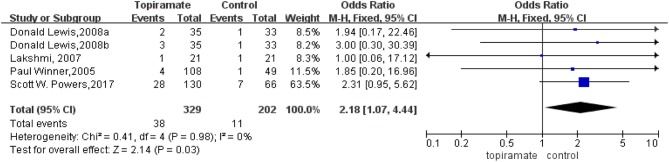
Withdrawals for any reason between topiramate and placebo groups.

All studies mentioned side effects and adverse reactions. The overall incidence of adverse events was more frequent in topiramate-treated group than that in placebo. Serious adverse event like suicide attempt was only reported in one incidence treated with topiramate. Adverse events occurring more frequently in topiramate group than that in placebo included paresthesia, loss of weight, upper respiratory tract infection, paresthesia, anorexia, fatigue, and so on (Figure 6). Numbers of withdrawals for any reason in the topiramate group significantly increased than those in the placebo group (*n* = 531; 95% CI, 1.07–4.44; *Z* = 2.14; *P* = 0.03) with low heterogeneity (*I*^2^ = 0%; *P* = 0.98) (Figure 7). We then carried out the meta-analysis of each common side effect that reported in the trials. As shown in Table 2, weight decrease (*n* = 395; 95% CI, 2.73–22.98; *Z* = 3.81, *P* < 0.01) and paresthesia (*n* = 531; 95% CI, 3.05–13.18; *Z* = 4.94; *P* < 0.01) significantly increased in patients with topiramate.

**Table 2:** Side effects and adverse reactions of topiramate vs. placebo.

**Side effects**	**Topiramate placebo No. of events/No. of participants**		**Relative risk (95% CI)**	***Z***	***P***	**Heterogeneity**		
						***Z***	***df***	***I*^**2**^%**
Loss of weight	39/259	5/136	7.92 (2.73–22.98)	3.81	<0.01	3.97	2	50
Paresthesia	71/329	8/202	6.34 (3.05–13.18)	4.94	<0.01	0.79	4	0
Upper respiratory tract infection	54/308	24/181	1.37 (0.82–2.31)	1.19	0.23	2.81	3	0
Abdominal pain	22/199	12/136	1.24 (0.60–2.57)	0.58	0.56	2.35	3	0
Fatigue	48/308	20/181	1.37 (0.78–2.41)	1.09	0.28	4.43	3	32
Anorexia	26/199	9/136	2.12 (0.94–4.75)	1.82	0.07	0.60	3	0
Injury	17/308	16/181	0.62 (0.31–1.26)	1.31	0.19	5.26	3	43

## Discussion

Topiramate became the first and only drug approved by FDA for migraine prevention in children of 12–17 years old in 2014. Although studies have proved that topiramate can reduce migraine frequency and improve quality of life in adults (17), evidence for topiramate to prevent migraine in children and adolescent was insufficient. A meta-analysis published in 2017 found that topiramate failed to decrease the number or increase 50% response rate. However, there were several serious defects in this analysis (as was mentioned in *Introduction*). In this study, we have corrected these flaws and evaluated the efficacy and safety of topiramate in the prevention of pediatric migraine. The results demonstrated that topiramate had a significant advantage in reducing the migraine days and PedMIDAS scores than placebo; however, it still failed to increase 50% response rate. It meant that topiramate could significantly decrease headache days and migraine-related burden. In the included studies, three trials (23–25) found that the decrease in monthly migraine days in the topiramate group was significantly greater compared with the placebo group. As for response rate, one study showed ≥50% response rate favored topiramate at 100 mg/day, not 50 mg/day (25). In the two studies in which patients were treated with topiramate at 2–3 mg/kg/day, one (23) found that topiramate achieved statistical significance in ≥50% responder rate. However, the other study (24) revealed ≥75% responder rate, rather than the 50% responder rate, was significantly higher in topiramate group than the placebo group. There were two researches reporting PedMIDAS changes, and one found that patients treated with topiramate experienced significant decrease in the PedMIDAS scores (23). One study measuring school absenteeism reported that the decrease in school absenteeism was significant among topiramate-treated children (23). Numbers of acute analgesic medications were evaluated in only one study, and no significant difference was found between the two groups (23).

Of particular note is that in the Donald Lewis, research, different dosages (50 and 100 mg/day) of topiramate were studied (25). Donald Lewis found that topiramate at 100 mg/day, instead of 50 mg/day, resulted in a statistically significant reduction in monthly migraine days and a greater percentage of patients experiencing a ≥50% reduction. Although this paper made the conclusion that topiramate at 50 mg/day had no efficacy in the prevention of pediatric migraine, a double-blind, dose comparison study of topiramate demonstrated that in both the 25 and 100 mg/day topiramate-treated groups, headache days per month decreased significantly (26). There were 100% of 25-mg patients responding with a ≥50% reduction in migraine days and 71% of 100-mg patients, which implied that low dosage of topiramate could also help to prevent pediatric migraine. Thus, in our opinion, it is not appropriate to include only topiramate at 100 mg/day in the previous meta-analysis, which is probably the main reason for our different results.

Furthermore, there is also an RCT with topiramate at 50, 100, and 200 mg to prevent migraine in children (27). However, the trial used the median percentage reductions in monthly migraine days as the main outcome. It demonstrated that compared with placebo, topiramate at 100 and 200 mg/day could reduce median percentage reductions in migraine days. All dosage of topiramate failed to significantly decrease the days of acute medication use compared with placebo.

This study illustrated that there was no significant difference in the percentage of patients experiencing a ≥50% reduction in monthly headache days between topiramate and placebo groups. The result may be due to the high placebo response rate of children (28). While placebo effects have been predicted ~35% in migraine studies of adults, the placebo effects of pediatric migraine trials can reach to 50% or higher (29). In our study, the percentage of patients responding with a ≥50% reduction in migraine days was 50.50 vs. 59.27% in the topiramate group. The difference between topiramate and placebo groups was too small to demonstrate the drug efficacy (30).

Although topiramate was reported to be well-tolerated in most studies, our results showed that numbers of withdrawals were more in the topiramate group. Like other antiepileptic drugs, topiramate has many adverse events, and some of them were serious. Migraine patients seem to be more sensitive to topiramate-associated side effects than those with epilepsy (31). All the four included studies reported that the topiramate group was associated with higher rate than the placebo group. Most of the side effects were mild to moderate and seemed to be related with the dosage of topiramate (27). The most common side effects in this meta-analysis were paresthesia, weight loss, fatigue, somnolence, upper respiratory tract infection, memory impairment, aphasia, and cognitive disorder, which were similar with the previous clinical trials in adults and children (17, 32). Rare but serious suicide attempt was observed when patients were treated with topiramate other than placebo (33). In 2008, the association between suicidality and antiepileptic drugs (AEDs), especially topiramate, was issued by FDA. Screening for psychiatric comorbidities before and during the treatment is suggested in patients with topiramate.

There were also RCTs evaluating the efficiency of topiramate vs. other drugs. Powers et al. (22) also evaluated the efficiency of topiramate vs. amitriptyline for pediatric migraine. No difference was found between topiramate and amitriptyline in migraine days and headache-related disability. Ashrafi et al. (34) also reported that there was no statistically significant difference between topiramate and cinnarizine in preventing pediatric migraine. However, another RCT (35) showed that topiramate at 50 mg/day produced better efficacy to reduce monthly headache days compared with propranolol 80 mg/day.

This meta-analysis followed rigorous data extraction procedures and credible data for analysis, and the included studies were all with high quality and at low risk of bias. However, there are several limitations that must be addressed here. First, after rigorous screening, our analysis only included four papers including five studies, and one of them involved a relatively small sample size. Second, in this meta-analysis, some outcome measures showed significant heterogeneity, such as ≥50% reduction in monthly headache days (*I*^2^ = 71%) and PedMIDAS score (*I*^2^ = 59%). However, only five trials were included in this analysis, and no variables could explain it. Third, migraine had a relative long course and chronic tendency for both children and adults, so a longer treatment duration than 12–20 weeks was reported in the included papers. Thus, the optimal therapeutic response and long-term drug efficacy should be evaluated further. Finally, there were only three measuring indexes in our results. Indexes evaluating quality of life and the use of analgesic medications were reported in few studies. More useful efficacy parameters should be measured and reported as recommended by the IHS.

## Conclusion

In conclusion, the current evidence demonstrates that topiramate shows greater beneficial effects for the prophylaxis of pediatric migraine than placebo. It can significantly decrease monthly headache days and migraine-related burden in migraine patients <18 years old. However, it failed to increase 50% response rate. Adverse events seem to be more frequent in topiramate-treated children. As for the limitations of the present study, more high-quality placebo-controlled RCTs are needed.

## Data Availability Statement

All datasets generated for this study are included in the article/supplementary material.

## Author Contributions

XWu: study conception, design and organization, acquisition of data, analysis and interpretation of data, drafting of the manuscript, critical revision of the manuscript for important intellectual content, statistical analysis, administrative, technical, material support, and study supervision. YZ: study conception, design, organization, and acquisition of data. ML: acquisition of data, analysis and interpretation of data, and statistical analysis. XYu: acquisition of data and analysis and interpretation of data. XYe: acquisition of data, and analysis and interpretation of data. XWa: study conception, design and organization, acquisition of data, analysis of data, critical revision of the manuscript for important intellectual content, administrative, technical, and material support, and study supervision. PS: acquisition of data, analysis and interpretation of data, statistical analysis, administrative, technical, and material support, and study supervision.

### Conflict of Interest

The authors declare that the research was conducted in the absence of any commercial or financial relationships that could be construed as a potential conflict of interest.
